# Recent advances on fermentation of mustard plant (*Brassica juncea* L.): microbial community, fermentation processing and sensorial quality: a review

**DOI:** 10.3389/fmicb.2026.1784857

**Published:** 2026-03-10

**Authors:** Nan Diao, Angye Cai, Yongtong Zhou, Baijun Long, Zhen Mo, Siwei Shang, Yumeng Liu, Jiaxin Xu, Wenzhong Hu, Ke Feng, Sarengaowa

**Affiliations:** 1 College of Life Science, Zhuhai College of Science and Technology, Zhuhai, Guangdong, China; 2 Faculty of Medicine, Macau University of Science and Technology, Macau, Macao SAR, China

**Keywords:** *Brassica juncea* L., fermentation processing, fermented mustard plant, microbial community, sensorial quality

## Abstract

Mustard (*Brassica juncea* L.), rich in vitamins, minerals, and glucosinolates, yields fermented products valued for their distinct flavor and health benefits, particularly across East and Southeast Asia. The fermentation process is primarily driven by a complex microbial community dominated by lactic acid bacteria (LAB) such as *Lactobacillus fermentum*, *Lactobacillus pentosus*, and *Lactobacillus plantarum*. These microbes metabolize substrates to generate organic acids, volatile compounds, and free amino acids, which collectively shape the product’s flavor and sensory quality. This review systematically summarizes recent progress in mustard fermentation, focusing on: the composition, succession, and function of microbial communities across different regions and fermentation stages and their influence on fermentation characteristics; the regulatory effects of key processing parameters—including fermentation vessel, temperature, and salt concentration—on microbial ecology, metabolic pathways, and final product quality; the chemical basis of taste attributes such as sourness, umami, bitterness, and pungency alongside the formation and evolution of aroma compounds during fermentation, and their links to microbial metabolism and biochemical pathways like glycolysis and the tricarboxylic acid cycle; and the formation patterns of potential risk factors such as biogenic amines and nitrite during fermentation, along with strategies to control their levels through process optimization and starter culture selection. Finally, future research directions are outlined, emphasizing the integration of omics and synthetic biology technologies to elucidate flavor formation mechanisms, develop stable starter cultures, and establish standardized processes. These advances aim to achieve consistent flavor, improved quality, and safe production of fermented mustard products, supporting the sustainable development of the industry.

## 1 Introduction

Mustard plant (*Brassica juncea* L.), an annual or biennial herbaceous plant of the Brassicaceae family, is an important vegetable crop widely planted and consumed worldwide. It not only has a profound history of cultivation in China but is also widely distributed in Japan, India, Africa, Central Asia, and Southeast Asia, having become an important part of global food culture (Fan et al., 2017). Mustard plants are further categorized into four major groups—root mustard, stem mustard, mustard greens, and mustard seed—which together encompass 16 varieties and several varietal types (Kang et al., 2021). Fermentation is a widely practiced and accepted ancient technique for food storage (Caplice and Fitzgerald, 1999), originating from advanced biotechnological techniques, such as osmosis via salting, microbial fermentation, and enzyme-mediated protein degradation, leading to biochemical changes in the vegetable (Rhee et al., 2011). Fermented foods have received a rising degree of attention, primarily due to their associated nutritional and health advantages (Mukherjee et al., 2022). Among these foods, fermented vegetables are widely favored thanks to their rich nutrients and flavors and prolonged shelf life (Xiao et al., 2018). Generally, fresh mustard greens are typically fermented in brine, where microbial metabolism produces organic acids, free amino acids, and volatile compounds, creating a distinctive flavor. During this process, lactic acid bacteria and other microbes degrade macro molecules, lowering pH while generating various flavor components. Fermentation duration, salinity, and other factors collectively shape the microbial community, ultimately determining the product’s flavor profile (Luo et al., 2021; Yang et al., 2020). Fermented mustard green, is popular in China due to its unique flavor, crispy texture, delicious taste, and health benefits (Xiao et al., 2020), and it is the soul of some dishes (e.g., Chinese sauerkraut fish and pickled mustard noodle). In China, the annual production of fermented mustard plant reaches 5.2 million tons, creating a market value of over 1.2 billion dollars. Mustard is valued not only for its distinctive flavor but also for its rich nutritional composition. Leaf mustard (*Brassica juncea*) contains approximately 0.8–1.5% carbohydrates, 1–2% protein, 0.13–0.4% fat, along with abundant vitamins (e.g., vitamin C, vitamin K) and minerals (e.g., calcium, iron, potassium) (Deng et al., 2010; Kim et al., 2007). These components serve as essential substrates for fermentation. Microbial metabolism during fermentation transforms these constituents into complex flavor compounds. For instance, secondary fermentation—whether spontaneous or inoculated—applied to salted mustard substantially alters the composition and concentration of volatile components. Studies indicate that secondary fermentation typically increases the variety and total content of key flavor compounds such as esters and alcohols (e.g., ester content can rise from about 31 to 38%), while reducing acid levels, collectively shaping the more intense and balanced characteristic flavor of fermented mustard (Chao, 2015). Thus, fermentation functions not merely as a preservation method but as a core bioprocessing step that profoundly enhances the flavor and quality of mustard.

However, at present, fermented leaf mustard is mainly obtained by spontaneous fermentation. Briefly, fresh leaf mustard is washed and dried, then immersed in brine (4–8% NaCl, w/w) and fermented by the microorganisms that happen to be present at ambient temperature for 10–15 days (Ge et al., 2021), which causes the quality of fermented leaf mustard to vary greatly. For the production of superior fermented mustard green products, two key aspects are critical: first, a thorough understanding of their sensory and nutritional qualities; second, exploration of microbial interactions and their contributions to quality attribute formation during product processing.

Producing high-quality fermented mustard requires a comprehensive investigation of microbial community dynamics, fermentation processing, sensory quality, and safety control. To this end, this review synthesizes recent advances by focusing on four interrelated core dimensions: Microbial Community Dynamics elucidates the composition, succession, and functional roles of microbial consortia across different regions and fermentation stages, with emphasis on the dominant lactic acid bacteria, yeasts, and their interactions driving the fermentation process; Fermentation Processing and Control evaluates the effects of key parameters—such as fermentation vessel, temperature, and salt concentration—on microbial ecology, metabolic pathways, and final product characteristics; Sensorial Quality and Flavor Formation clarifies the biochemical basis underlying the characteristic sour, umami, bitter, and pungent tastes as well as the complex aroma, texture, and color of fermented mustard, linking microbial activities and metabolic pathways such as glycolysis and the TCA cycle to specific sensory attributes; Safety Control Strategies examines the formation mechanisms of major safety concerns including biogenic amines and nitrite, summarizing strategies to mitigate these risks through process optimization and starter culture selection. By integrating knowledge across these areas, this review aims to provide a scientific foundation for optimizing fermentation processes, improving product consistency and sensory quality, ensuring safety, and guiding future research toward the sustainable development of the fermented mustard industry. The following sections present a detailed analysis of each key aspect.

## 2 Microbial community

The process of fermenting mustard plant involves a variety of microorganisms and is accompanied by complex microbial community dynamics that drive complex changes in material composition, ultimately forming the product’s quality. In the most common natural fermentation processes, differences in the dominant microbial communities in different regions, variations in the fermentation conditions caused by different pickling methods, and the length of the fermentation time all affect microbial composition and sensory characteristics of fermented vegetables. Therefore, in order to obtain high-quality products, it is necessary to determine the dominant strains in the fermentation process of mustard plant.

### 2.1 Types of microorganisms in fermented mustard plant from different regions

Fermented mustard plant, a traditional fermented food with a venerable history, are cherished for their distinctive taste and flavor profiles. However, due to regional differences and changes in the fermentation process, the microbial composition of mustard showed diversity. This microbial diversity affects the sensory properties and quality of fermented mustard, as well as its safety and potential health benefits.

Microbial community analysis of traditional fermented mustard green reveals substantial biogeographic specificity. Nguyen et al. (2013) characterized 881 lactic acid bacteria (LAB) isolates from 21 Vietnamese fermented mustard green samples, identifying *Lactobacillus fermentum* (56.6%), *Lactobacillus pentosus* (24.4%), and *Lactobacillus plantarum* (17.1%) as dominant taxa, alongside rare occurrences of *Pediococcus pentosaceus* (1.0%) and *Lactobacillus brevis* (0.5%). Contrastingly, Thai Phak-gard-dong fermentation systems exhibit a predominance of *Bacillus weihenstephanensis* (Tamang, 2009), while Himalayan Goyang fermentations maintain a core microbiota of *Lactobacillus* spp. supplemented by *Pediococcus*, *Lactococcus*, *Pentososus*, *Pichia*, and *Rhodotorula* yeasts (Tamang, 2009; Shah and Singhal, 2017). Regional divergence persists in Chinese fermentations: Zhejiang mustard brines harbor *Firmicutes* (81.73%) and *Proteobacteria* (17.61%), with Lactobacillus (54.67%) and *Vibrio* (14.39%) being key genera (Zhang J. M. et al., 2021). Northeastern Chinese cabbage demonstrates similar phylum-level distribution but distinct genus composition (*Pantoea, Weissella, Pseudomonas*) (Chang et al., 2022). PCR-DGGE (Polymerase Chain Reaction-Denaturing Gradient Gel Electrophoresis) analysis of Sichuan paocai (China) showed that Lactobacillus and uncultured bacteria are the dominant microbial communities during the fermentation process. In addition, there are also Lactococcus and Pediococcus, but their proportions are relatively small. The bacterial diversity is higher than that of Yibin paocai and Nanchong winter vegetables (Zhang, 2012). Metagenomic sequencing corroborates *Firmicutes* (73.8%), *Proteobacteria* (17.5%), and *Ascomycota* (8.7%) dominance, featuring *Weissella* (44.3%) and Lactobacillus (26.8%) (Mi et al., 2022). Notably, Yibin yacai exhibits *Lactobacillus predominance* (50% total bacteria), with 6/12 sequenced strains representing uncultured taxa, suggesting *Lactobacillus lactis* dominance (Zhang, 2012).

Fungal communities, though less diverse than bacterial consortia, exert critical functional influences. In Sichuan Province, China, fermentations display *Hanseniaspora uvarum* dominance alongside substantial uncultured fungal populations (Zhang, 2012). Late-stage fermentation yeasts (*Candida magnoliae*, *Kodamaea ohmeri*) critically modulate flavor profiles and storage stability (Demain and Adrio, 2008). This biogeographic patterning of microbial consortia—Southeast Asian Lactobacillus-centric communities versus Korean *Lactobacillus brevis*/*Lactobacillus sakei* predominance (Lee et al., 2006; Lee et al., 2005), contrasted with China’s Firmicutes-diverse systems—directly correlates with distinct organoleptic properties in regional fermented mustard plant. Crucially, low-abundance taxa (e.g., yeasts regulating esterification pathways, *Pseudomonas* influencing redox balance) engage in complex metabolic cross-talk with dominant bacteria, collectively determining fermentation kinetics and end-product quality (Zhang, 2012, Mi et al., 2022, Demain and Adrio, 2008). Results are compiled in Table 1.

**Table 1:** Influence of geography and time on the changes in microbial species and quantity in mustard-pickled vegetables.

Region	Fermented vegetable	Raw materials	Fermentation time	Microbial species	Dominant genera of microorganisms	References
Vietnam	Duamuoi Camuoi	cải bẹ	/	*Lactobacillus fermentum, Lactobacillus pentosus, Lactobacillus plantarum, Pediococcus pentosaceus*	*Lactobacillus, Pediococcus* (microorganisms)	Nguyen et al. (2013)
Thailand	Phak-gard-dong	Karen, Lawa, and Shan, three types of local mustard greens	/	*Lactobacillus, Candida albicans, Clostridium welchii*, *Pediococcus genus, Bacillus weihenstephanensis*	*Lactobacillus, Pediococcus, Bacillus* (microorganisms)	Yongsawas et al. (2022), Tamang (2009)
Korea	Kimchi	Chinese cabbage, mustard greens, scallions, young radish	Early stage: *Enterococcus faecalis, Lactobacillus acidophilus subspecies* Mid stage: *Lactobacillus zeae* Late stage: *Lactobacillus plantarum, Lactobacillus casei*	*Enterococcus faecalis, Lactobacillus acidophilus subspecies, Lactobacillus plantarum, Lactobacillus casei, Lactobacillus zeae, Candida albicans, Vibrio* spp.*, Pediococcus* spp.*, Lactobacillus brevis*, *Lactobacillus sakei*	*Lactobacillus, Pediococcus* (microorganisms), *Yeast genus* (fungi)	Shah and Singhal (2017), Jeong et al. (2013)
China	Sichuan paocai (SCP)	Cabbage, mustard plant	/	*Lactic acid bacteria* (LAB)*, acetic acid bacteria, Omerkoda yeast, Hansenula Debarry yeas*t, *Aspergillus Candida tropicalis, Meyerozyma guilliermondii*	*Lactobacillus, halophilic bacteria* (microorganisms), *yeast genus* (fungi)	Zhang (2012)
China	Yibin Yacai	Mustard root	/	LAB, *halophilic bacteria, acidophilic bacteria, Hanseniaspora uvarum*	*Lactobacillus, halophilic bacteria* (microorganisms), *yeast genus* (fungi)	Zou et al. (2022)
China	Zhacai	Mustard root	Initial stage: *Leuconostoc mesenteroides* Mid-to-late stages: *Lactobacillus plantarum, Lactobacillus brevis*	*Streptococcus thermophilus, Lactobacillus plantarum, Lactobacillus brevis*	*Lactobacillus* (microorganisms)	/
China	Northeastern mustard sauerkraut	Mustard greens	Early stage: *Streptococcus subsp., Lactobacillus taiwanensis, Lactobacillus acidophilus, Lactobacillus fermentum, Lactobacillus subsp., Lactobacillus gasseri, Lactobacillus plantarum, Lactobacillus johnsonii* Mid stage: *Lactobacillus fermentum, Lactobacillus subsp., Lactobacillus curvatus, Lactobacillus plantarum* Late stage: *Lactobacillus paracasei subsp. paracasei*	*Lactobacillus plantarum, Lactobacillus brevis, Lactobacillus reuteri, Lactobacillus acidophilus*	*Lactobacillus* (microorganisms)	Wu et al. (2015)

### 2.2 Microbial dynamics in mustard plant during fermentation

At the beginning of fermentation, a diverse array of microorganisms coexist. However, as the fermentation proceeds, the metabolites produced by the dominant flora inhibit the growth of other microorganisms, resulting in a gradual decrease in microbial diversity (Tamang, 2009; Zhang J. M. et al., 2021). For instance, during the early fermentation of fermented vegetables like kimchi and Chinese fermented mustard plant, microorganisms like *Candida*, *Enterococcus*, *Pseudomonas*, and Lactobacillus can be detected (Shah and Singhal, 2017). These microorganisms produce acid and carbon dioxide, which rapidly lowers the pH, thereby inhibiting the growth of harmful microorganisms (Shah and Singhal, 2017). These microorganisms produce acid and carbon dioxide, which rapidly lowers the pH, thereby inhibiting the growth of harmful microorganisms (Shah and Singhal, 2017). Additionally, in the fermentation process of Korean water kimchi made from mustard leaves, *Leuconostoc* not only becomes the dominant bacterium in the initial stage—metabolizing to lower the environmental pH and thereby laying the foundation for subsequent fermentation—but also maintains its dominant status throughout the entire fermentation cycle, acting as the core microbial driver for the formation of the characteristic flavor of Korean water kimchi (Pederson and Albury, 1961, Bi et al., 2000, Jeong et al., 2013). In the initial stages, some pathogenic and spoilage microorganisms, such as *Sphingomonas*, *Pseudomonas*, as well as certain anaerobic bacteria, may also be present. However, these microorganisms disappear quickly, likely due to niche selection (He et al., 2017).

As the fermentation process progresses, the microbial community exhibits significant stage-specific succession, with the intermediate stage being a critical node for microbial community restructuring and functional transformation. In the fermentation of mustard plants into kimchi, this process manifests as a transition from the atypical lactic acid fermentation stage to the facultative anaerobic lactic acid bacteria-dominated stage: initially, lactic acid cocci utilize residual oxygen for atypical fermentation, and as oxygen is consumed, facultative anaerobic lactic acid bacteria take over (Zhang, 2012, Li, 2015, Jing, 2016), driving the fermentation toward a stable acidification stage. Similarly, *Lactococcus lactis subsp.* is one of the key components of the core microbial community during the intermediate stage of kimchi fermentation in northeastern China. As a facultative anaerobic lactic acid bacteria, it adapts to the low-oxygen and low-pH environment of kimchi fermentation, becoming a mid-term driver of the region’s kimchi flavor formation (Wu et al., 2015).

When the fermentation enters a later stage, Lactobacillus becomes the absolute significant flora, with *Lactobacillus plantarum* and *Lactobacillus casei* being particularly prominent (Shah and Singhal, 2017). In Northeastern China’s mustard sauerkraut, homolactic acid bacteria dominate, and the dominant species is *Lactobacillus coryniformis* subsp. torquens (Li, 2015; Wu et al., 2015), while in kimchi fermented with mustard as the raw material, *Lactobacillus plantarum* and *Lactobacillus casei* are the main strains (Xiong et al., 2012). In fermented mustard plant from Guangyuan (Sichuan Province, China), LAB constitute 74% of the total flora, with *Lactobacillus fermentum* accounting for 62.16% and *Lactobacillus delbrueckii* for 27.63% (Liu A. P. et al., 2017). In the later period of mustard fermentation, the populations of *Lactobacillus plantarum*, *Lactobacillus sakei*, *Lactobacillus brevis*, and *Lactobacillus parabuchneri* increase significantly (Shah and Singhal, 2017). Furthermore, microbial diversity decreases markedly in the late fermentation stage. Common flora include *Weissella, Leuconostoc, Lactococcus*, and Lactobacillus (Zhou et al., 2022).

Microbiologic population changes in the fermentation process of various vegetables follow a similar pattern: *Enterococcus* and *Leuconostoc* dominate in the early stages; *lactic acid cocci* and *facultative anaerobic* LAB take over in the middle stages; and homolactic acid bacteria, such as *Lactobacillus plantarum* and *Lactobacillus casei*, emerge as dominant in the final stages. Different fermentation stages reveal that the acid production of microorganisms, the consumption of oxygen in the fermentation process, and the changing pH environment are the key driving factors for the succession of microorganisms in the vegetable fermentation ecosystem. Results are compiled in Table 1.

### 2.3 Fermentation processing

During the production process of fermented mustard plant, the fermentation technique is one of the key factors to determine the quality of the fermented mustard plant. The unique textures and flavors largely depend on the choice of fermentation technique. This section reviews the fermentation techniques of fermented mustard plant and other fermented vegetables, hoping to provide a reference for subsequent research on the fermentation process of fermented mustard plant.

### 2.4 Types of microorganisms in different kinds of mustard plants

Different types of mustard exhibit variations in their morphological structures, and the types of microorganisms attached to their surfaces also differ, and these microorganisms mainly originate from the endogenous bacterial communities in the raw materials. The fermented mustard green known as Dưa Muối, produced in the northern plains of Vietnam, is made from the leaves of *Brassica juncea*, a mustard species native to Asia. During the fermentation process, *Lactobacillus fermentum* emerges as the dominant strain, constituting 56.6% of the total microbial population, followed by *Lactobacillus pentosus* at 24.4% and *Lactobacillus plantarum* at 17.1% (Nguyen et al., 2013). In contrast, *Pediococcus pentosaceus* and *Lactobacillus brevis* are present in lower quantities, accounting for 1.0 and 0.5% of the total LAB isolates, respectively. Additionally, trace amounts of other strains—each contributing 0.1%—have been identified, including *Lactobacillus paracasei*, *Lactobacillus panis*, and *Lactococcus lactis*. Collectively, these LAB produce organic acids and carbon dioxide, which quickly lower the pH of the environment. This acidification effectively suppresses the proliferation of harmful microorganisms. Furthermore, the high acidity generated by these initial dominant species and subsequent LAB not only suppresses their own overgrowth but also facilitates replacement by more acid-tolerant LAB, such as *Lactobacillus plantarum*, which ultimately drives the fermentation to completion (Nguyen et al., 2013). In Japan, the sunki product, made from fermented red beet leaves, is dominated by bacterial strains including *Lactobacillus delbrueckii*, *Lactobacillus plantarum*, and *Lactobacillus fermentum*. The acidic pH environment and lactic acid produced by these LAB through their metabolism may exert a preservative effect on sunki by inhibiting the growth of spoilage microorganisms (Endo et al., 2008). China’s Yibin Yacai is a fermented product made from the stems and roots of mustard plants, but it distinguishes itself by using a unique small-leaf mustard variety native to the Yibin region, which is characterized by its elastic roots when mature. The primary fermenting microorganisms involved in Yibin Yacai production are LAB and *halophilic bacteria*. These microbial communities play a critical role in significantly increasing the content of amino acid substances and volatile flavor components in the final product. Among these volatile compounds, olefins (such as β-Myrcene and Valencene) and esters (including Isoamyl benzoate) stand out as key contributors to its distinct aroma and flavor profile (Zou et al., 2022). Phak-gard-dong, a fermented mustard green product from Thailand, relies on the natural endogenous and exogenous bacteria present on or within the mustard leaves to drive its fermentation. Notably, *Weissella* bacteria emerge as the most prevalent microbial group during this process, and these strains are particularly adept at producing bacteriocins—compounds that play a key role in extending the shelf life of Phak-gard-dong (Yongsawas et al., 2022).

**Figure 1:**
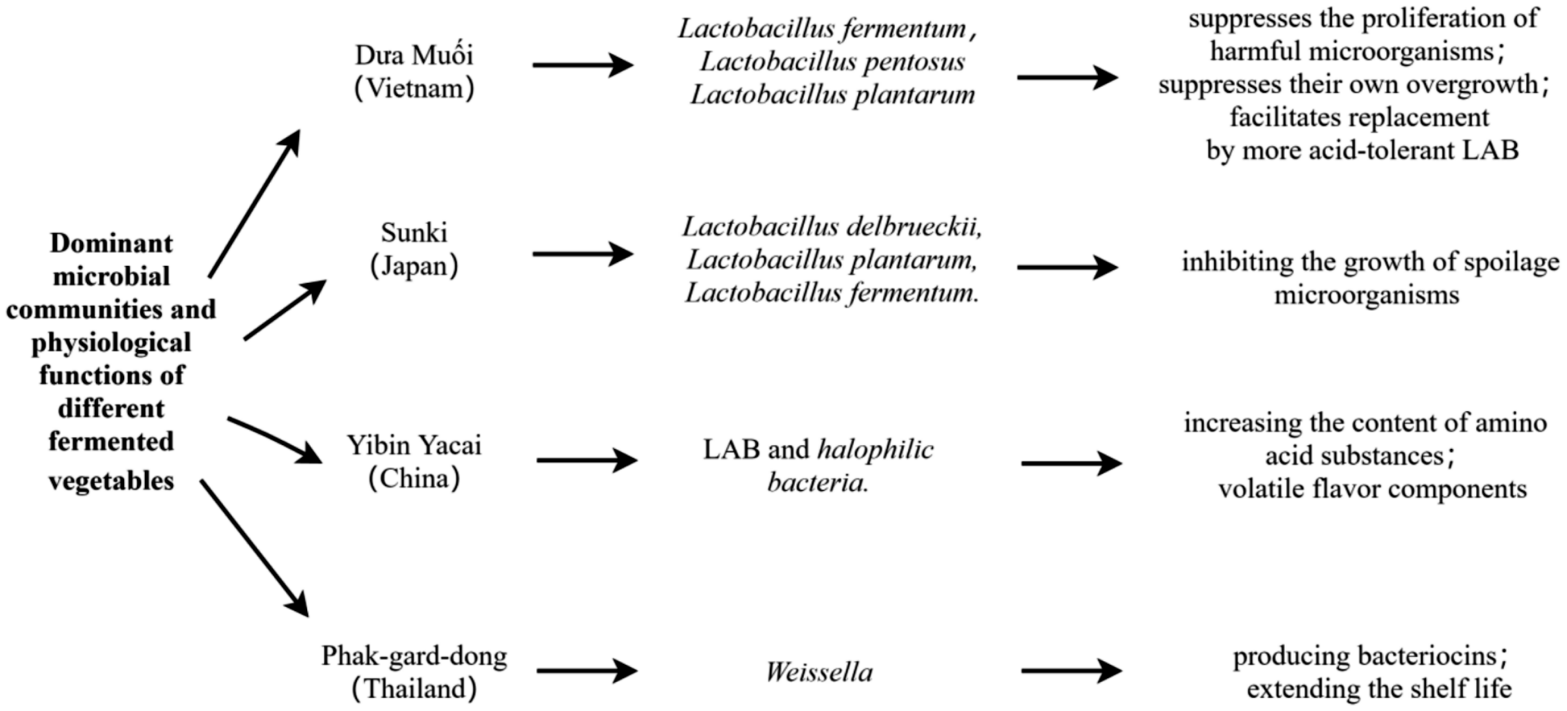
Dominant microbial communities and physiological functions of different fermented vegetables.

In conclusion, the fermentation of mustard plant and related Brassica species across various Asian cultures demonstrates a remarkable interplay between plant morphology and microbial ecology. Despite regional variations in the specific plant parts utilized (leaves, stems, or roots) and the resulting fermented products (Duamuoi, kimchi, sunki, Phak-gard-dong, or Yibin Yacai), LAB—particularly Lactobacillus species—consistently emerge as the dominant microbial drivers of the fermentation process. This universal predominance of LAB, including key species such as *Lactobacillus fermentum*, *Lactobacillus pentosus*, and *Lactobacillus plantarum*, underscores their vital role in shaping the biochemical and sensory attributes of fermented mustard plant products. Results are compiled in Figure 1.

### 2.5 Different fermentation environments of mustard plant

To date, there has been no specific research on the environment for fermented mustard plant. The material of the fermentation container, temperature, and the ratio of salt concentration are key parameters that directly influence the quality of the product. In view of this, this section systematically integrates the mature experiences of other pickled vegetable fermentation systems to provide a theoretical basis for establishing a precise fermentation model for mustard plant.

The main function of a fermentation vessel is to provide a suitable environment for microorganisms so that they can effectively carry out metabolic activities, thereby completing the fermentation process. During fermentation, the conditions within the vessel (e.g., pH value, temperature and oxygen supply) can be controlled to meet the requirements of a specific fermentation process. Additionally, fermentation vessels can prevent external contamination, ensuring the purity and stability of the fermentation process. Han et al. studied how container type affects kimchi fermentation by comparing traditional Onggi vessels (TOV), plastic airtight Onggi (PAOV), plastic covered (PCV), and plastic airtight covered (PACV) containers. Samples were taken every 48 h during the study, and on the tenth day, initial LAB counts (1.09 × 10^8^ CFU/mL) rose significantly, with TOV having the highest final count (1.42 × 10^10^ CFU/mL), with the authors noting the dominance of five strains: *Bacillus subtilis*, *B. licheniformis*, *B. safensis, Lactobacillus brevis*, and *B. pumilus* (Han et al., 2013). Meanwhile, a comparison of pH values across different containers (TOV pH 3.75, PAOV pH 3.92, PCV pH 3.89, PACV pH 3.99) revealed that when using TOV as the fermentation vessel, the pickling liquid achieved the lowest pH value (Han et al., 2013). The study confirmed that the distinctive structural design and material composition of TOV optimize probiotic growth during fermentation, particularly enhancing *Lactobacillus brevis*, at the same time, it can effectively inhibit the reproduction of harmful microorganisms. Notably, the organic acids generated via the enzymatic activity of lactic acid bacteria reached the highest levels among the four tested fermentation vessels, resulting in more pronounced umami and sour flavor profiles. These combined attributes render TOV superior to airtight plastic containers for traditional kimchi fermentation (Han et al., 2013).

**Figure 2:**
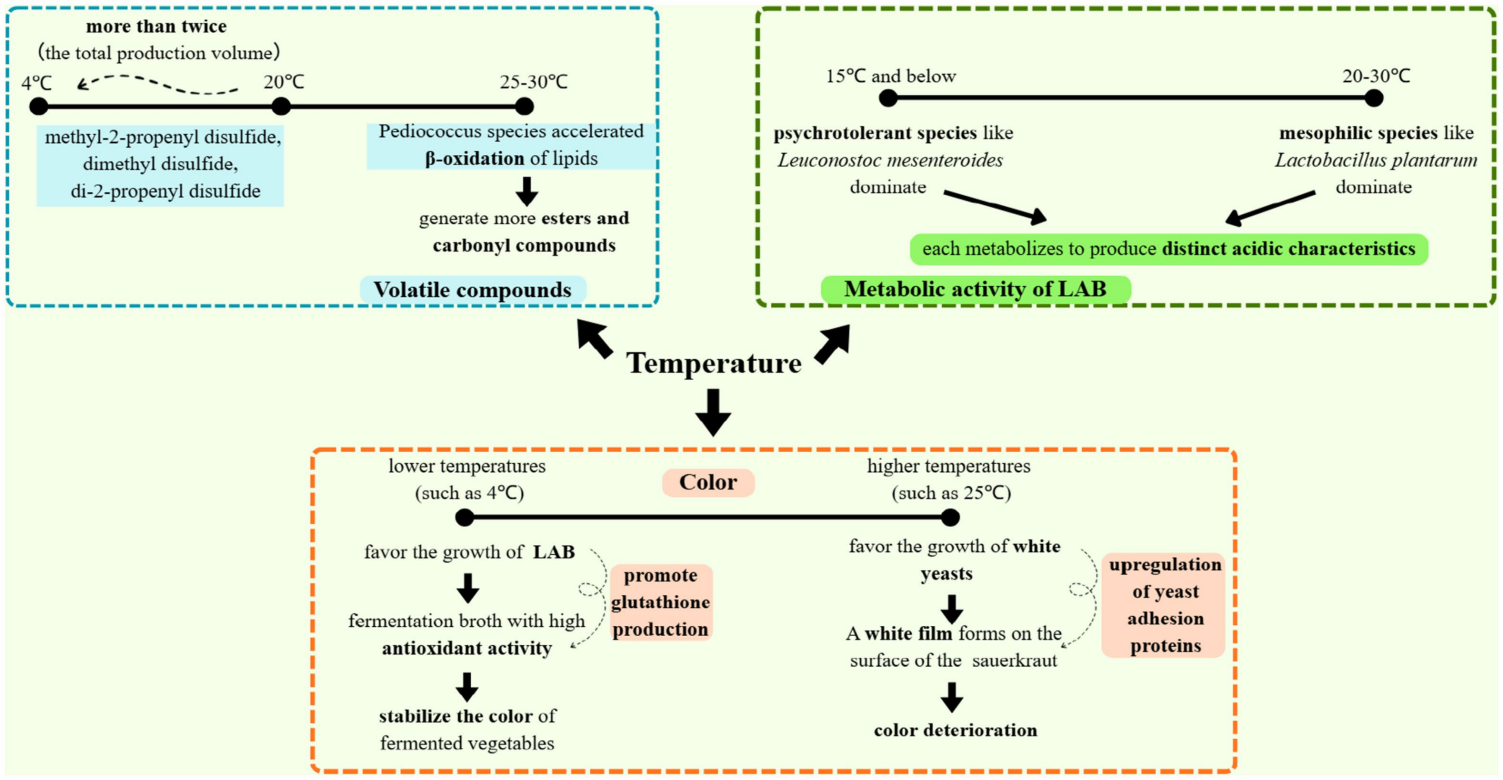
The multidimensional influence of temperature on the fermentation process.

Temperature is one of the key factors controlling the fermentation process, and microorganisms have their own optimal temperature range for growth. For instance, LAB such as *Lactobacillus plantarum* exhibit maximal metabolic activity between 20 and 30 °C, whereas psychrotolerant species like *Leuconostoc mesenteroides* dominate below 15 °C, producing distinct acid profiles (Hong et al., 2016). By regulating temperature, the growth of microorganisms can be influenced, thereby controlling the generation of volatile flavor compounds. Hong et al. found that the optimal maturation periods for mustard sauerkraut fermented at 4 °C and 20 °C were 35 days and 2 days, respectively, and that the amount of volatile compounds (e.g., methyl-2-propenyl disulfide, dimethyl disulfide, and di-2-propenyl disulfide) produced at 20 °C was more than double that at 4 °C (Hong et al., 2016). This temperature-dependent volatile synthesis correlates with the enhanced activity of microbial cysteine sulfoxide lyases. He et al. also found that the types of volatile flavor compounds in fermented vegetables increase with the rise in fermentation temperature (He et al., 2020). At higher temperatures (25–30 °C), *Pediococcus* species generate more esters and carbonyl compounds through accelerated β-oxidation of lipids. Fermentation temperature also affects the color of fermented vegetables. On the one hand, temperature can affect the rate of chemical reactions during fermentation, thereby influencing the formation of color; on the other hand, temperature indirectly affects the color through its impact on the microbial community structure: fermentation at lower temperatures favors the growth of LAB, whose fermentation broth has high antioxidant activity (e.g., glutathione production) and can stabilize the color of fermented vegetables (Lu et al., 2019). Suzuki et al. prepared fermented sauerkraut at four different temperatures and found that, at 4 °C, the sauerkraut had good color, but at higher temperatures (10, 15, 25 °C), white yeasts (*Debaryomyces hansenii*) grew rapidly, spreading to the surface of the sauerkraut and forming a white film, leading to color deterioration. This biofilm formation correlates with the temperature-induced upregulation of yeast adhesion proteins. This indicates that fermentation temperature is one of the critical factors controlling the discoloration of sauerkraut (Suzuki et al., 2018). In summary, temperature serves as a critical control parameter in vegetable fermentation, governing microbial behavior, metabolic outputs, and sensory attributes (flavor and color) through interconnected biological and chemical mechanisms. Results are compiled in Figure 2.

**Figure 3:**
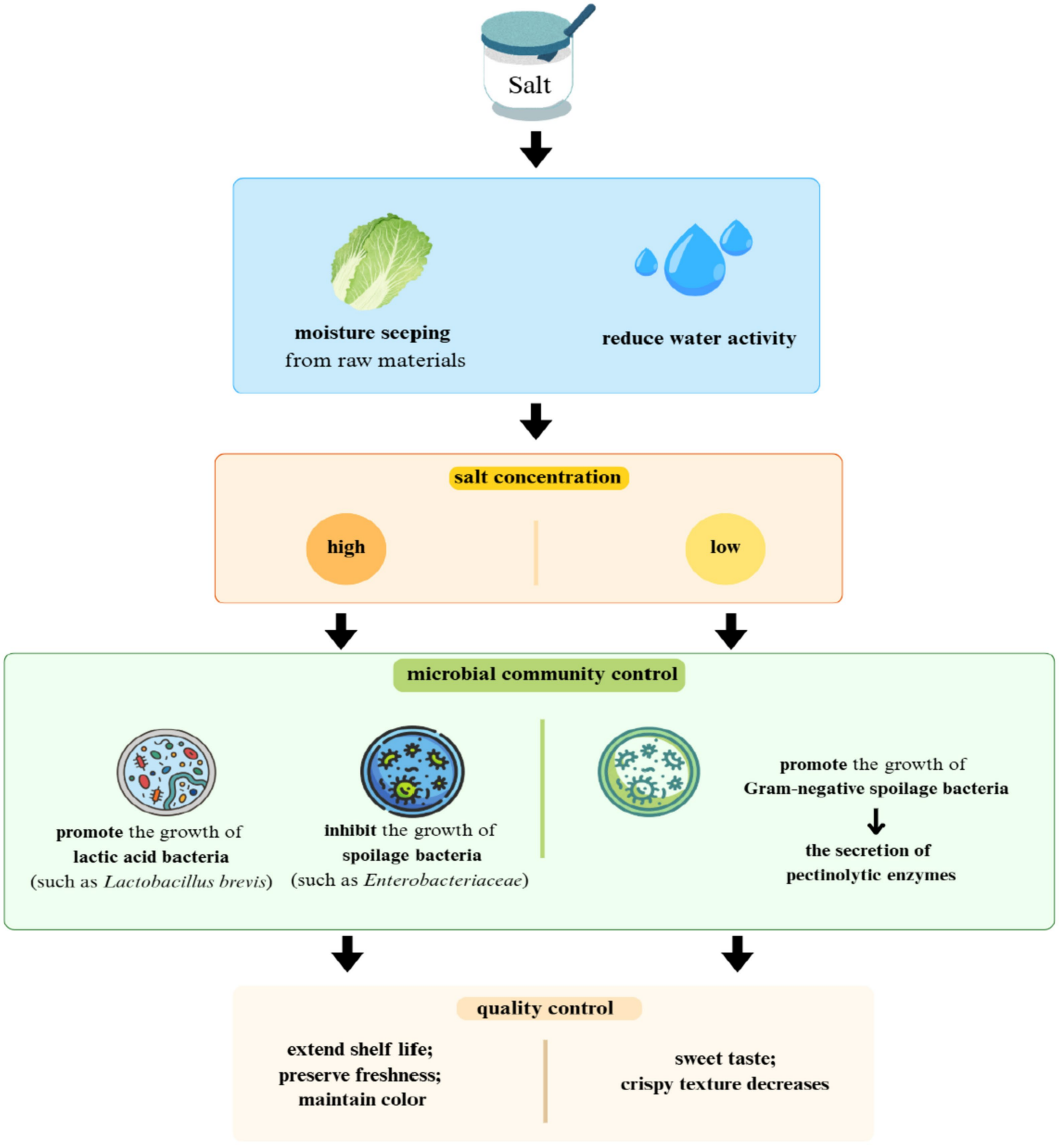
The multidimensional influence of salt on the fermentation process.

Salt is essential in fermenting vegetables, and different salt concentrations can directly affect the quality of the fermented product. Fermentation occurs in the presence of brine, dry salt, or sugar. These help to leach out moisture from the raw materials and act as a medium for the thriving microbial community. Salt and sugar substances enhance the flavor and can also help to control the growth of spoilage bacteria by reducing the water activity (Shah and Singhal, 2017). Generally, the salt concentration for fermented mustard plant is around 4%, but the amount of salt used can vary for different fermented vegetable products. Specifically, the brine used for fermenting vegetables in Vietnam is made from salt and sugar, and the amounts of salt and sugar can vary from family to family, resulting in different flavors (Nguyen et al., 2013). Caper berries (*Capparis spinosa* L.), primarily fermented in the Mediterranean and exported globally, undergo a commercial process involving 4–7 days of water immersion, followed by 10–12% brine to complete fermentation. The salt concentration is adjustable: it is increased to 15% (*in situ*) for enhanced preservation or replaced for large-scale storage pre-commercialization. Fermentation of different Capparis species (e.g., *C. ovata*) is modulated by brine concentration and LAB; 15% fresh brine, for instance, preserves optimal color and quality in *C. ovata* (Shah and Singhal, 2017). During the processing of fermented chili peppers in Europe, maintaining a brine concentration of 10% can effectively extend the product’s shelf life (Shah and Singhal, 2017). Similarly, in China, during the winter or early spring, mustard is fermented in the presence of 8–10% salt, which inhibits the growth of microorganisms such as *Enterobacteriaceae* and *Bacillus*, while promoting the growth of beneficial fermentation strains such as *Lactobacillus mesenteroides*, *Lactobacillus brevis*, and *Lactobacillus plantarum* (Shah and Singhal, 2017). A higher salt concentration also inhibits the growth of yeast (Zhang et al., 2023). Salt plays a crucial role in enhancing taste and mouthfeel of pickles and, in one study, the salt content was reduced to 57% of the total salt content, which is 0.5%. This resulted in a sweet pickle with reduced acidity; however, a lower concentration of salt promotes the growth of Gram-negative spoilage bacteria, whose pectinolytic enzymes can cause the pickles to lose their crisp texture (Shah and Singhal, 2017). Results are compiled in Figure 3.

In summary, the fermentation vessel primarily determines the physical ecology of fermentation, temperature serves as the switch for microbial metabolic activities, and salt regulates microbial community structure and flavor intensity. These three factors collectively shape microbial diversity from different perspectives, ultimately encoding diverse sensory characteristics. This holds significant reference value for achieving a balance between the quality and safety of fermented mustard plants.

## 3 Sensory quality

As a traditional pickled vegetable, fermented mustard plants possess unique sensory characteristics. Well-fermented mustard plants should exhibit a harmonious balance of sourness and umami. However, fresh mustard plants contain a special natural compound called glucosinolates, which convert into sulfur-containing compounds like allyl isothiocyanate during fermentation, imparting bitter and spicy flavors. Additionally, high-quality mustard plants should develop a rich, characteristic fermented aroma. Different varieties may display distinct fragrance profiles, such as floral-fruit or almond notes. Although some off-flavors may occur during fermentation, these typically diminish or disappear as the process concludes. In terms of texture, premium mustard greens should offer crisp or tender mouthfeel—the higher the crispness, the better the overall experience. Regarding color, traditional fermented vegetables often appear yellowish-brown or yellowish-green, while well-fermented products tend to have a bright, glossy yellow hue.

Generally, researchers systematically evaluate the sensory quality of mustard greens using methods such as quantitative descriptive analysis (QDA) and sensory radar charts (Liu et al., 2025) (Table 2). Sensory evaluation typically requires the formation of a specialized sensory evaluation team to analyze various sensory characteristics based on established scoring criteria, with different studies employing varying scoring systems. For instance, Piasecka-Józwiak et al. (2013) used a 6-point scoring scale according to the PN-ISO 4121:1998 standard to assess the sensory quality of products fermented for 7 days, with evaluation indicators including color, aroma, taste, and texture. They also employed a 9-point pleasure scale to evaluate overall acceptability. In contrast, Liu et al. (2025) conducted a preference test for sensory evaluation. The reviewers scored the color, texture, flavor, taste, and overall acceptability of fermented mustard leaves using a 10-point pleasure scale, with results visually presented in the form of sensory radar charts.

**Table 2:** Sensory evaluation detection index and method.

Sensory characteristics	Monitoring indicators	Sensory evaluation methods	References
Sensory evaluation	Taste, color, texture, flavor, overall acceptability	Quantitative descriptive analysis (QDA)	Piasecka-Józwiak et al. (2013), Chen et al. (2025)
		Sensory radar charts	Liu et al. (2025)
Taste	pH value	pH meter	Liu et al. (2021), Yu et al. (2022a,b)
	TA content	Titration method	
	Non-volatile substances (e.g., organic acids, free amino acids, etc.)	HPLC	Zhang C. C. et al. (2021), Zhang J. M. et al. (2021), Zou et al. (2022)
Odor	Volatile compounds	GC–MS; HS-SPME	Yu et al. (2022a,b), Li et al. (2025), Zhong et al. (2022)
Texture	Mustard stem, mustard leaves	TA XTplus texture analyzer; TA-XT2i texture analyzer	Ge et al. (2021), Zhao et al. (2022), Sarengaowa et al. (2024), Zhang et al. (2019)
Color	Mustard stem, mustard leaves	HunterLab digital color difference spectrophotometer; CR-400 lab mini colorimeter; CR-410 lab mini colorimeter	Tinpovong et al. (2024), Zhang et al. (2019), Sarengaowa et al. (2024)

### 3.1 Taste

**Figure 4:**
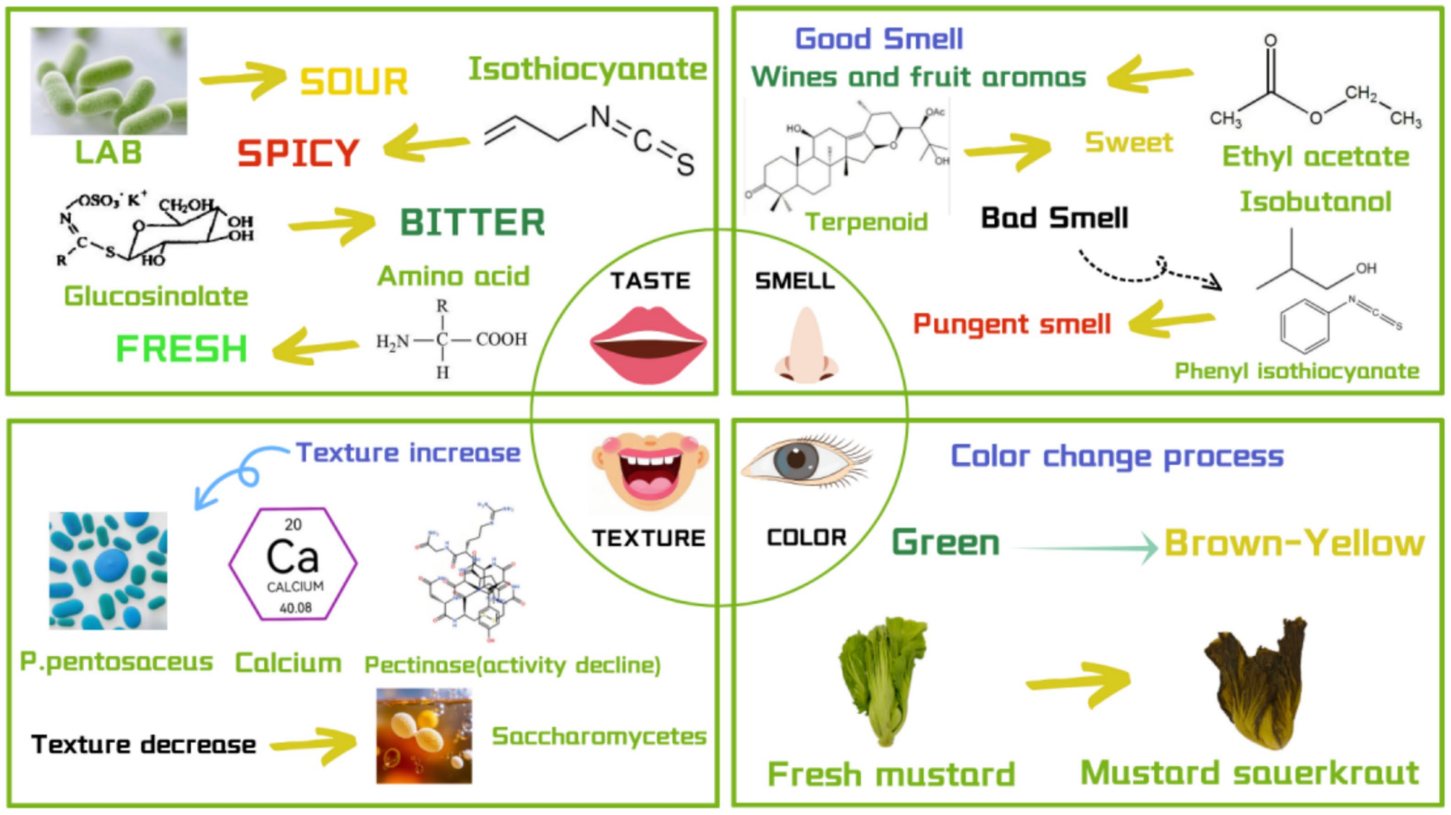
Sensory analysis diagram of fermented mustard green.

The flavor characteristics of fermented mustard plant mainly include sour, spicy, bitter, and umami. Research has indicated that the sour characteristic is primarily influenced by changes in pH and TA, while the spicy characteristic results from sulfur compounds produced during fermentation. The bitter and umami characteristics are mainly caused by the interaction of various amino acids with different flavors. These four flavors interact to create the unique flavor profile of fermented mustard plant (Figure 4).

#### 3.1.1 Sour

pH and Titratable Acidity (TA) are important chemical indicators related to product maturity in fermented vegetables. Generally speaking, when the pH value is lower than 4.0 and the TA is higher than 0.3 g/100 g, the fermented vegetables are considered to have reached maturity (Zhang C. C. et al., 2021). In common detection methods, pH is typically measured directly using a pH meter, while total acid content is usually analyzed by titration method (Hou et al., 2023) (Table 2). Through our research, we found that scholars have achieved rich research results on fermented mustard plant in Hangzhou City, Yibin City, Guangzhou City, Zhejiang Province, Sichuan Province and Guangdong Province, which may be because the fermented mustard plant products in these three regions are relatively common. In Liu et al.’s (2021) study, employed a pH meter and titration method to measure pH and total acid content, respectively. They observed that in the Hangzhou region, the pH value dropped below 4.0 after 90 days of fermentation, while the total acid content increased progressively during the fermentation process. Similarly, Yu et al. (2022b) employed the same detection method and found that the fermentation cycle in Guangzhou was relatively short, with the pH value dropping to approximately 3.5 after just 15 days of fermentation. The total acid (TA) content showed an overall upward trend during the early fermentation stage. Comparative studies revealed that in Hangzhou, Yibin, and Guangzhou, the pH values of fermented mustard greens in these three regions exhibited a pattern of initial slow decline, followed by sharp fluctuations, and ultimately stabilized as fermentation progressed (Liu et al., 2021). In contrast, the TA value of mustard greens showed a consistent upward trend. The findings of Kong et al. (2025) and Zhang C. C. et al. (2021) are largely consistent with this conclusion. Therefore, we believe that the ripening time of fermented mustard plant is different in different regions, but the change trend of pH values and TA values is similar.

The decrease in pH is driven primarily by the production of organic acids during fermentation (Yu et al., 2022b). As the number of LAB increases, their organic acid content also increases. This is because lactic acid is converted into various organic acids through the action of different enzymes during fermentation. This process provides the final product of fermented mustard plants with a rich and unique flavor. For the detection of non-volatile substances (e.g., organic acids, free amino acids, etc.), high-performance liquid chromatography (HPLC) is currently the primary method (Table 2). Zhang C. C. et al. (2021) employed HPLC to identify organic acids in fermented mustard from Hangzhou, including lactic acid, acetic acid, malic acid, succinic acid, citric acid, and fumaric acid. Among these acids, malic acid is the main organic acid; lactic acid and acetic acid are the main acid products, while the quantities of succinic acid, citric acid, and fumaric acid are relatively low during the fermentation process of fermented mustard plants. With increasing fermentation time, lactic acid and acetic acid showed the most pronounced increase in concentration, citrate acid decreased, succinate acid increased, and fumaric acid concentration did not change much during fermentation (Zhang C. C. et al., 2021). Zou et al. (2022) also employed HPLC to specifically detect seven typical organic acids in the Yibin region, including oxalic acid, tartaric acid, malic acid, lactic acid, acetic acid, citric acid, and succinic acid. The difference lies in the predominance of oxalic acid, citric acid, and succinic acid, while the content of acetic acid was relatively lower. Yu et al. (2022b) identified multiple organic acids, including lactic acid, acetic acid, citric acid, malic acid, oxalic acid, fumaric acid, and succinic acid, in fermented mustard samples from Guangzhou. During the initial fermentation phase, oxalic acid was the predominant organic acid, with its concentration showing a slight decline throughout the process. The levels of lactic acid and acetic acid experienced a sharp increase during the mid-fermentation stage before stabilizing. Notably, the concentrations of malic acid and citric acid exhibited a minor increase in the first 6 days before declining, as these acids are typically converted into lactic acid and acetic acid by lactic acid bacteria. It is worth mentioning that the contents of fumaric acid and succinic acid gradually decreased throughout the fermentation process.

#### 3.1.2 Spicy

In fresh mustard plant, the pungency primarily originates from their roots. During the fermentation process of fermented mustard plant (in addition to producing the organic acids mentioned earlier) numerous compounds are generated, including isothiocyanates, nitriles, aldehydes, esters, alcohols, ketones, hydrocarbons, and sulfur-containing compounds (Liu et al., 2021). The compounds responsible for the pungent flavor mainly consist of sulfur-containing compounds (isothiocyanates and rhodanates) and nitriles. These contribute to the formation of spicy, garlic-like, and root-like flavors in fermented mustard plant. Studies have shown that allyl isothiocyanate is the primary source of the pungent taste in mustard plant (Li et al., 2025). Derived from cruciferous vegetables, this unique flavor component, comprised of black mustard green glycosides, imparts distinctive aromatic characteristics (Zhao et al., 2007). Most of the nitrile components in fermented mustard plant originate from plant materials, though some also stem from microbial and enzymatic processes (Mutanen and Pajari, 2011). However, research has revealed that allyl isothiocyanate content gradually decreases during fermentation, attributed to various enzymatic reactions occurring during this process. As its concentration diminishes, the distinctive pungent, garlic, and root-like odors also diminish (Liu et al., 2025). In summary, both fresh mustard plant and fermented mustard plant contain sulfur compounds, but the substances influencing their pungent flavors differ. Among these, allyl isothiocyanate serves as the primary component responsible for the pungent flavor in fermented mustard plant.

#### 3.1.3 Bitter

Research has indicated that the primary components responsible for the bitter taste in fermented mustard plant are glucosinolates and their hydrolysis products, along with bitter amino acids. Glucosinolates, a natural compound found abundantly in cruciferous plants, undergo enzymatic hydrolysis during fermentation to produce bitter compounds like isothiocyanates, which impart the distinctive bitterness of mustard (Yu et al., 2022b; Yang et al., 2023). Liu et al. demonstrate that as fermentation progresses, both isothiocyanate and glucosinolate concentrations decrease (Liu et al., 2021). Beyond volatile compounds, amino acids—key substrates for aromatic compound formation—emerge during fermentation. Bitter amino acids include tyrosine (Tyr), leucine (Leu), isoleucine (Ile), valine (Val), phenylalanine (Phe), lysine (Lys), histidine (His), arginine (Arg), and methionine (Met) (Liu et al., 2023). Wang et al.’s (2024) research reveals that bitter fatty acids (FAAs) levels slightly increase after 2–3 days of fermentation, enhancing the bitter flavor profile. This finding is similar to that of Liu et al. (2023), in their study of fermented chili peppers, suggested that the increase in bitter amino acids may be due to microbial metabolism, resulting in an unpleasant taste.

#### 3.1.4 Umami

Amino acids contribute significantly to the development of umami flavor in fermented mustard plant, and the free amino acids (FAAs) provide different tastes. Taste-active amino acids include bitter amino acids (tyrosine, leucine, isoleucine, valine, phenylalanine, lysine, histidine, and arginine), sweet amino acids (threonine, serine, glycine, alanine, and methionine), and umami amino acids (glutamic acid and aspartic acid) (Liu et al., 2023; Wang et al., 2024). The literature indicates that the level of umami free amino acids (FAAs) rose markedly in the initial phase (days 1–2) and continued to rise in the subsequent phase (days 2–3). This may be because glutamic acid and aspartic acid in mustard were gradually released after being decomposed by microbial proteases in the fermentation environment, so the FAAs increased and enhanced the umami flavor of fermented mustard plant (Wang et al., 2024). In conclusion, both glutamic acid and aspartic acid are crucial amino acids for providing the umami flavor, with glutamic acid showing particularly abundant content during fermentation. This conclusion is similar to the results of Zhang C. C. et al. (2021) and Yu et al. (2022b).

### 3.2 Odor

The flavor profile of mustard greens is primarily determined by both volatile and non-volatile compounds. Free volatile compounds can be directly detected through human olfaction, whereas bound aromatic compounds (e.g., glycoside ligand-containing compounds) require chemical analysis methods for detection (Wang et al., 2024). To accurately identify flavor components in fermented mustard greens, most experiments employ analytical techniques such as gas chromatography–mass spectrometry (GC–MS) and headspace-solid phase microextraction (HS-SPME) (Table 2).

#### 3.2.1 Good odor

In the fermentation process, In addition to the non-volatile compounds (e.g., organic acids and amino acids), volatile compounds, including hydrocarbons, alcohols, aldehydes, ketones, esters/ethers, and heterocyclic compounds, and sulfur-containing substances are detected. These components collectively shape the unique flavor profile and texture of mustard-pickled vegetables (Xiao et al., 2018). Most volatile compounds impart a pleasant aroma to fermented mustard plant. Li et al. (2025) employed the HS-SPME method to extract volatile compounds from pickled vegetable samples, revealing that all samples collected from various cities in Guizhou Province contained phenylethanol. This compound can be utilized as an edible flavoring agent with almond-like aroma, representing the simplest aromatic alcohol. Similarly, benzyl alcohol was detected in Sichuan Province’s preserved vegetables, showing increased content during fermentation. However, it gives fermented mustard plant a honey and rose petal fragrance (Zhang et al., 2023). Aldehydes constitute a crucial determinant of pickled vegetables’ unique flavor, categorized into saturated and unsaturated types. Generally, unsaturated aldehydes impart cheese and fruity aromas (Adams et al., 2008). Studies indicate that benzenepropanal contributes to malt-like aromas, whereas octaldehyde contributes sweet orange notes (Li et al., 2025). In fermented foods, low concentrations of benzaldehyde impart an almond-like aroma, while coexisting with other aldehydes or esters confers a fruity and sweet scent to fermented mustard plant (Chu and Yaylayan, 2008). Another study revealed 2-nonenone, a common ketone in fermented foods, which provides cream and fruity notes, giving fermented mustard plant a subtle, light-bodied taste (Wang and Arntfield, 2014). The synthesis of ethyl acetate from lactic acid (of bacterial origin) and ethanol (of yeast origin) contributes a mild alcoholic and fruity aroma to fermented mustard plant (Zhang et al., 2023). In the study by Xiao et al. (2020), GC–MS was employed to analyze volatile compounds in the samples, identifying ethyl nonanoate with a waxy aroma, which is typically formed by the reaction of nonanoic acid with ethanol. Additionally, during the fermentation process of mustard plant, octanoic acid, pyrrole compounds, and terpenoids were identified (Zhao et al., 2007, Yang et al., 2023, Zhong et al., 2022). Octanoic acid is an acidic compound with fruit and caramel aromas (Akamatsu et al., 2021). Studies show that pyrrole compounds are mainly generated via the catabolic breakdown of amino acids (Xiao et al., 2020), serving as characteristic flavor compounds in chestnut-scented green tea that impart a nutty aroma and subtle fruity notes to mustard plant (Yang et al., 2023). Furthermore, among volatile compounds in traditionally fermented vegetables, scholars have discovered camphor alcohol, a terpene derived from the acetate pathway, which endows mustard greens with a pleasant floral fragrance and is abundant in mustard plant (Xiao et al., 2020; Zhong et al., 2022).

#### 3.2.2 Bad odor

The volatile compounds detected in fermented vegetables do not simply contribute pleasant flavors and enhance people’s appetites; some compounds can also cause an unpleasant odor. In fermented mustard plant, thioglycosides not only taste bitter but also emit an unpleasant smell similar to sulfur (Yang et al., 2023). In addition, allyl isothiocyanate and phenyl ethyl isothiocyanate emit a strong, green, irritating odor, while butanol emits a weak, bitter allyl alcohol odor (Zhang et al., 2023). As mentioned above, aldehydes are classified into saturated aldehydes and unsaturated aldehydes. Unsaturated aldehydes possess a pleasant flavor, whereas saturated aldehydes exhibit a pungent and irritating taste (Adams et al., 2008). However, its bad odor is not only reflected in the fermentation process; the root stem of fresh mustard plant also has a pungent odor, and its seeds are applied in food processing to produce a pungent mustard condiment. Studies on the undesirable odor of fermented mustard plant are currently limited, and further studies will be required in the future.

In conclusion, volatile flavor compounds, including alcohols, esters, and phenols contribute fruity, sweet, green, and mushroom flavors, while some compounds, such as allyl isothiocyanate, phenylethyl isothiocyanate, and isobutytanol, contribute unpleasant flavors. Figure 4 presents the aromatic profile of fermented mustard plant. Therefore, volatile compounds need to be distinguished for future studies.

### 3.3 Texture

Texture, as the first consumer evaluation index in tasting food, plays an important role in consumer choice. A proper, crisp texture without unwelcome softening is attractive to consumers. Therefore, it is important to study texture changes in fermented mustard plant. The texture of plant tissue is a complex characteristic, and the main influencing factors are the integrity of pectin content and cell wall microstructure (Yang et al., 2023). The factors affecting the texture of vegetable tissue are shown in Figure 4. The defects in the hardness of vegetables largely depend on the activity level of pectinase (Ge et al., 2021). When the activity of pectinase decreases, the hardness of vegetables can remain stable (Yang et al., 2023). Under acidic conditions, the pectin in the mustard cell wall is gradually hydrolyzed, leading to the softening of the mustard plant. Pectinase secreted from fermented microorganisms also has a hydrolytic effect on pectin (Sarengaowa et al., 2024). Generally speaking, researchers mainly use TA XTplus texture analyzer and TA-XT2i texture analyzer to evaluate the texture of fermented mustard (Ge et al., 2021, Zhao et al., 2022, Sarengaowa et al., 2024) (Table 2). In Hangzhou, Zhang et al. (2019) used the TA XTplus texture analyzer to determine that the hardness of mustard samples was higher than that of the unfermented state (day 0) during fermentation (months 1, 2, and 3), and gradually decreased with the extension of fermentation time. The general fermentation period of fermented mustard plant in Thailand is 20 days; during the whole fermentation process, the hardness and friability values steadily decrease. This indicates that the average hardness of natural fermentation decreases over time (Tinpovong et al., 2024), which will have an impact on the texture of the sample. These findings provide some insight into maintaining the good taste of mustard cabbage. In conclusion, fermented mustard plant at home and abroad has been noted to have similarities in terms of their texture changes, and fermentation time is the main factor affecting the texture, as the longer the fermentation time, the more the overall hardness will decrease. The texture change in mustard plays a vital role in the natural fermentation process, which not only affects the sensory quality of mustard plant but is also related to its nutritional value and other flavor characteristics. It is an aspect of the mustard plant fermentation process that cannot be ignored.

### 3.4 Color

As one of the sensory evaluation criteria, color differences may affect consumers’ perception and acceptance of food. Color changes are mainly related to the values of L * (brightness), a * (red/green), and b * (yellow/blue) (Tinpovong et al., 2024). In terms of detecting chromatic aberration, researchers usually first observe the color change in the fermentation process of mustard and sauerkraut with the naked eye, and then use a color difference meter to determine the color value of specific parts (Table 2). In the common fermentation process, mustard plant gradually changes from green to yellow (Figure 4) (Paramithiotis et al., 2022). One study found that the color change in mustard is closely related to its fermentation mechanism. First of all, the fermentation process is affected by acidic conditions, the inhibition of chlorophyll enzymes, and the decrease in chlorophyll and carotenoid content (Yilmaz and Gökmen, 2016), resulting in a decrease in color intensity and the yellow color is enhanced, the whole gradually changes to yellow-brown. Next, by detecting the color of mustard sample through HunterLab digital color difference spectrophotometer, it was found that the brightness of PMG increased in the early stage of fermentation (5–10 days), mainly showing green. Intermediate fermentation (after 15 days) saw a peak in brightness (Tinpovong et al., 2024). The brightness enhancement may be attributed to the enzymatic browning reaction, which is catalyzed by enzyme polyphenol oxidase (PPO) activity (Yilmaz and Gökmen, 2016). In the later fermentation stage (20 days), the brightness value shows a downward trend, and the green chromaticity gradually turns to yellow. The mustard plant was determined to be edible when it had completely turned yellowish brown.

In the Hangzhou area, Zhang et al. (2019) used CR-400 lab mini colorimeter to determine the color difference value of the three-month fermentation period of mustard plant and found that the L * value (brightness) and the b * value (yellow) of the samples on day 0 were the highest, while the L * value and the b * value in the first, second, and third months of fermentation gradually decreased, and the color gradually faded. The experiment of Sarengaowa et al. (2024) used CR-400 lab mini colorimeter to determine the sample of mustard plant during the half-month fermentation period in Guangdong also produced the same result. Compared with the domestic samples of mustard plant and pickled cabbage, the foreign samples produced different results. In Chiang Mai, Thailand, where the fermentation time was about 20 days, with the increase of the fermentation period, the L* value (brightness) of the sample gradually increases, and the a * value (green) showed a significant increase, which proved that the green tone had shifted to other color components. At the beginning, the b * value (yellow) also increased with the fermentation time, and the b * value was the highest reached a peak after 15 days, but after 20 days, the b * value began to decline, and the yellow color gradually darkened (Tinpovong et al., 2024). Overall, it has been shown that during the fermentation process, the fermented mustard plant color changes from green to yellow, and the yellow color is most pronounced after 15 days of fermentation. By comparing the color change process of fermented mustard plant samples in different regions, we learned that the fermentation time is different in different regions, but the color change in fermented mustard plant is much the same. To summarize, color is not only an important factor affecting consumer choice; it is also one of the criteria through which we evaluate the maturity of mustard fermentation and edibility. It is a key part of the industrial production of fermented mustard plant products.

### 3.5 Formation mechanism

#### 3.5.1 Glycolysis

According to the literature, carbohydrates (such as sucrose, glucose, maltose, etc.) are metabolized through glycolysis to produce a series of intermediate metabolites, including glyceraldehyde-3-phosphate, pyruvate, phosphoenolpyruvate, and acetyl-CoA. Glyceraldehyde-3-phosphate can be further converted to cysteine, and sulfites (such as diallyl disulfide) are produced through sulfur metabolism pathways such as cysteine and methionine metabolism, which contribute unique aroma profiles in fermented vegetables. As a key intermediate product of glycolysis, pyruvate can be converted into lactic acid and acetic acid through the pyruvate metabolic pathway, which affects the flavor of fermented mustard plant during fermentation. Phosphoenolpyruvate can enter the aromatic amino acid metabolism pathway and be converted into phenylalanine, which is further metabolized to form phenylalanine acid and other compounds, which may give fermented products special aromatic properties. In addition, acetyl-CoA is converted to butyric acid, propionic acid, and ethanol through butyric acid metabolism, propionic acid metabolism, and pyruvate metabolism (Wang et al., 2023). The organic matter produced by these metabolic processes provides the flavor of fermented mustard plant.

#### 3.5.2 Tricarboxylic acids are destroyed

In the fermentation of mustard plant, LAB can produce acetic acid through pyruvate oxidase using acetyl-CoA as a substrate, and phosphate acetyltransferase and acetic acid kinase are also involved in this process. In addition, Halomonas, Halosporia, and Pseudomonas may convert acetyl-CoA and acetyladenyl adenylate to acetic acid by acetyl-CoA synthase (Wang et al., 2024). Malic acid, succinic acid, and citric acid are also involved in the citric acid cycle through the production and utilization of malic acid, which encodes enzyme genes mainly in the genomes of *halophilic bacteria* (Halomonas and Vibrio salt). Microorganisms produce various organic compounds through the tricarboxylic acid cycle, and the properties of different organic compounds will also produce various flavors.

#### 3.5.3 The role of microorganisms such as LAB

The spontaneous fermentation of mustard plant is a dynamic process involving a variety of microorganisms, with LAB playing a key role in shaping the aroma and flavor of fermented mustard green. In this literature review, it can be seen that the growth of LAB has an important effect on the changes in enzyme activity and chemical composition in the process of fermenting kimchi. Early stage of fermentation, the bacteria of the *Escherichia coli* family carried in the raw materials rapidly consumed oxygen in the environment, creating suitable conditions for the growth of facultative aerobic and heterotrophic LAB. After fermenting for 3 days, Streptococcus citrate and *Weissella* spp. became the dominant bacterial genera in the bacterial community, and C. citrate was the most abundant. With the progress of fermentation, homogeneous, fermented lactic acid bacteria dominated by Lactobacillus gradually dominated. Throughout the microbial community succession process, LAB decompose macromolecular substances (such as proteins) into small-molecule compounds (such as amino acids) by releasing a variety of enzymes, which further participate in the formation and evolution of flavor, ultimately giving kimchi its unique flavor characteristics (Zhu et al., 2024). In the process of fermenting mustard plant, LAB will also release a variety of enzymes to break down macromolecular substances into small molecules, which ultimately gives mustard sauerkraut a unique flavor profile.

**Figure 5:**
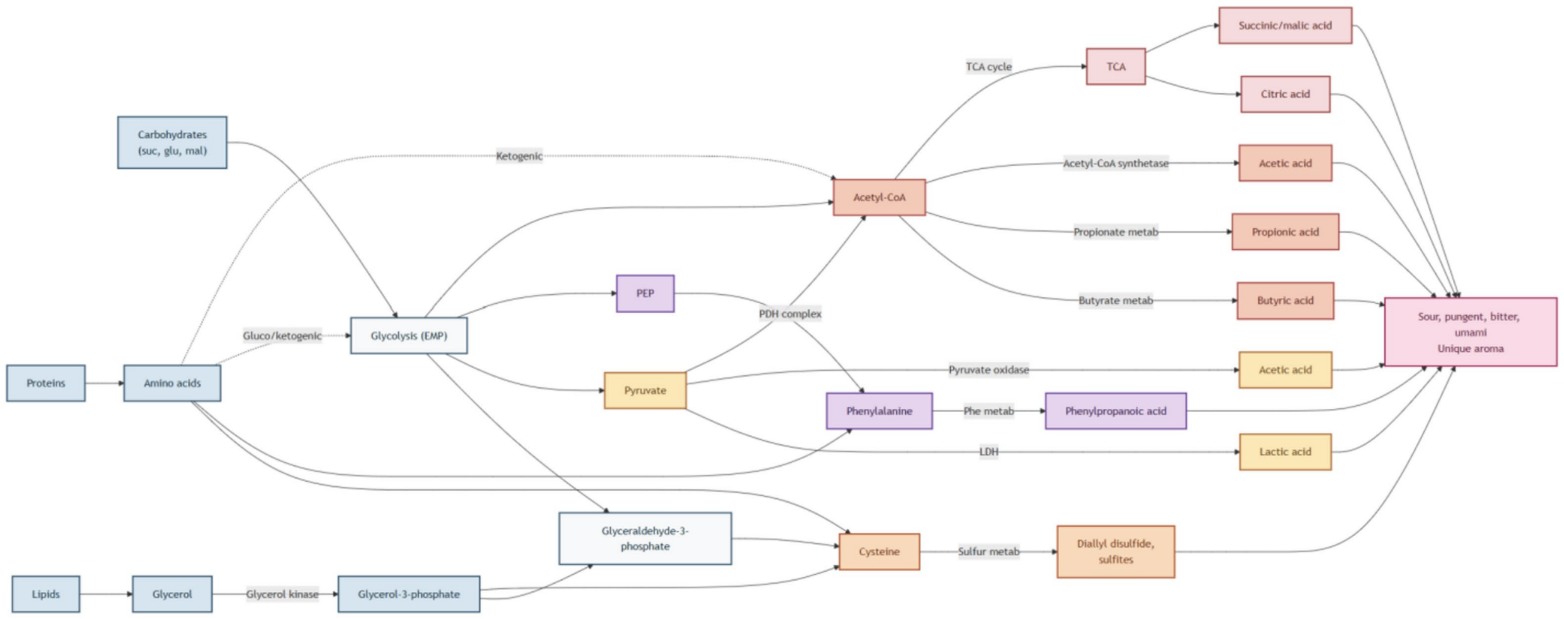
The flavor formation pathway of mustard greens and sauerkraut.

Through the mechanism of tricarboxylic acid deterioration, glycolysis, and microorganisms, fermented mustard plant is endowed with various flavor characteristics (e.g., sour, spicy, bitter, umami) (Figure 5).

### 3.6 Safety

#### 3.6.1 Biogenic amines

Biogenic amines is an organic nitride with small molecular content, and its generation mainly depends on the enzymatic decarboxylation reaction of amino acids by microorganisms during fermentation (Jin et al., 2019). Tryptamine, β-phenethylamine, putrescine, cadaverine, histamine, and tyramine are typical biological amines (Liu L. B. et al., 2017). Low doses of bioamines can achieve rapid metabolism in the intestine (Swider et al., 2020), while excessive doses of bioamines will induce rashes, headaches, hot flashes, nausea and other adverse symptoms, causing serious harm to human health (Feddern et al., 2019). Therefore, it is necessary to determine the concentration of biogenic amines. The fermentation process of mustard plant was tested, and the results showed that there were seven biological amines and their predecessors (tyrosine, tryptophan, histidine, lysine and arginine) (Li et al., 2018). Yu et al. (2022a) found that changes in biogenic amine precursors were mainly concentrated in the early fermentation phase (days 4–6); tyrosine, tryptophan, histidine, lysine, and arginine increased rapidly and subsequently remained relatively stable until the end of the fermentation process. Changes in biogenic amines occur throughout the entire fermentation process. As shown in the results of Li et al. (2018), only two very low concentrations of biogenic amines—cadaverine and putrescine—were detected at the beginning of the process. Subsequently, tryptamine, tyramine, histamine, cadaverine, and putrescine accumulated rapidly and tend to stabilize when the concentration reached a specific level (Yu et al., 2022a). Histamine is the most important biogenic amine in the fermentation process, and its concentration can account for half of the total amount of biogenic amines, followed by tyramine and β-phenythylamine (Tsai et al., 2005). Facts have proved that tyramine, tryptamine, histamine, cadaverine, and putrescine are the main biological amine components of fermented mustard plant. Biogenic amines can be used as an effective indicator for the detection of food corruption (Yu et al., 2022b). This may increase the risk of various health problems (Yu et al., 2021b). Therefore, the study and control of microorganisms that promote the production of biogenic amines during food fermentation is an area of great concern. Through comparing the physicochemical properties of Pa (green onion) kimchi and Gat (mustard leaf) kimchi, *L. brevis* was found to be the most abundant, and most biogenic amines (including β- phylethylamine, putrescine, and tyramine) had higher yields than other species, most likely due to the formation of biogenic amines (Tsai et al., 2005; Lee et al., 2019). Interestingly, inoculated and indigenous LAB strains significantly degraded both tryptamine and histamine. Interestingly, both inoculated LAB and naturally formed LAB show significant degradation ability for troptopanin and histamine. The results imply that the LAB strains, including *L. brevis*, may have the potential to degrade specific biogenic amines The results confirm that LAB, such as *L. brevis* may have the potential to degrade specific biological amines (Landete et al., 2007). This result was similar to that of a previous study by Yu et al. (2021a). This finding has some implications for the safety of fermented mustard plant and requires further research.

#### 3.6.2 Nitrite

Although vegetables are rich in various nutrients, they also contain harmful substances that cannot be ignored, including nitrates, nitrites, and heavy metal pollutants such as cadmium and lead (Wang et al., 2016). In particular, nitrite, which is usually formed and accumulated in fermented or pickled vegetables, often causes food safety issues and has been classified as a Group 2A carcinogen by the World Health Organization (Lee et al., 2019). Although nitrates themselves are not directly toxic, when nitrates are stored for a period of time or enter the human digestive system, they can be converted into nitrites and nitrosamines due to the action of nitrate reductase (NR) and bacteria. These compounds have serious risks with respect to teratogenicity, carcinogenicity, and mutagenicity (Zhao et al., 2011). Therefore, studying the changes of nitrite in the fermentation process is of positive significance to ensure product safety.

Similarly, nitrite is also very common in fermented mustard plant. A moderate amount of nitrite contributes to the fermented flavor and quality of fermented mustard plant, but excessive intake can not only increase the risk of diseases such as cancer, but also lead to hemoglobin oxidation (Wang et al., 2018). The nitrite content of pickled samples in Hangzhou increased first and then decreased, and its peak occurs in the middle stage of the fermentation process (Kato et al., 1987). Yibin sprouts (Yibin Yacai) in Sichuan province had a general fermentation period of 3 years, and a study found that the nitrite content was at its highest in the first year, then decreased over time (Shen et al., 2018). The fermentation period of fermented mustard plant samples in Guangzhou was 15 days, and the nitrite content increased rapidly during days 0–4, peaked on day 4, decreased during days 4–9, and gradually stabilized after day 9 (Zou et al., 2022). Based on the fermentation of fermented mustard plant in various regions of China, the results show that the change trend affected by fermentation time factors is basically consistent: the content of nitrite decreased with the increase in fermentation time (Yu et al., 2022b), and the concentration of nitrite will increase rapidly and peak during the early fermentation period (days 2–4) (Zou et al., 2022), exceeding the maximum content of nitrite in pickled vegetables stipulated in China’s Food Safety Law (20 mg/kg) (Nan et al., 2005). The rapid degradation of nitrite decreased in the middle of the fermentation process and further decreased during the later stage (i.e., after more than 10 days) to its lowest level (Zhong et al., 2022), meeting the national safety edible standards (Xu et al., 2019). Studies show that the nitrite content can be further reduced, and the quality and safety of fermented mustard plant can be optimized by screening the dominant fermentation strains and adjusting the curing process. Some LAB isolated from the fermentation system (mainly *Leuconostoc* spp.) have been proven to have the ability to reduce the content of nitrite (Zhang J. M. et al., 2021). The content of nitrite and the relative abundance of *Leuconostoc* show a positive correlation (Yu et al., 2021a), but nitrite exerts a negative effect on *Brevundimonas*, *Weissella*, *Bacillus*, and Acinetobacter. In particular, malate and tartaric acid can be used as nutrients to support the growth of Leuconostoc spp. and accelerate the degradation of nitrite, indicating that the activity of Leuconostoc spp. in fermented mustard plant can reduce the content of nitrite (Xu et al., 2019). Therefore, as one of the important factors affecting the fermentation safety of mustard plant, there is still ample room for further research into effectively reducing nitrite and ensuring food safety.

## 4 Conclusion

Fermented mustard plant is a traditional food with cultural and economic importance, valued for its sensory characteristics, nutritional benefits, and complex microbial ecology. This review examined key determinants of product quality, including microbial community dynamics, physicochemical transformations, flavor formation pathways, and safety-related factors. Sensory attributes such as sourness, spiciness, bitterness, and umami originate from organic acids, volatile compounds, and free amino acids generated during fermentation. Lactic acid bacteria dominance throughout the fermentation process drives flavor development and texture preservation. Safety concerns including biogenic amines and nitrite accumulation were also addressed; nitrite concentrations typically peak during early fermentation and decline thereafter, with risks effectively mitigated through optimized fermentation conditions and targeted strain selection.

Despite accumulated knowledge, spontaneous fermentation remains widely practiced and leads to inconsistent product quality, underscoring the need for controlled fermentation systems. Future research should prioritize four interconnected areas. First, precise microbial control requires the application of omics technologies—particularly metagenomics and metabolomics—to elucidate the metabolic networks and functional roles of dominant microorganisms. Second, flavor optimization can be advanced through gene editing and synthetic biology approaches to enhance biosynthesis of desirable volatile compounds while reducing off-flavor formation. Third, safety improvements depend on developing tailored starter cultures capable of minimizing hazardous byproducts such as biogenic amines and nitrite without compromising sensory integrity. Fourth, industrial standardization necessitates the establishment of robust, scalable fermentation protocols that ensure reproducible product quality across production batches. The integration of multi-omics analysis, synthetic biology tools, and precision fermentation platforms offers a coherent pathway toward significant improvements in product quality, safety profile, and consumer acceptance. This review systematically synthesizes current understanding of microbial succession, flavor formation mechanisms, and safety challenges associated with traditional mustard fermentation, thereby providing a scientific foundation and forward-looking perspective for continued research and technological innovation in fermented vegetable processing.
